# Comparative Analysis of Physiological, Hormonal and Transcriptomic Responses Reveal Mechanisms of Saline-Alkali Tolerance in Autotetraploid Rice (*Oryza sativa* L.)

**DOI:** 10.3390/ijms232416146

**Published:** 2022-12-18

**Authors:** Chunying Zhang, Weilong Meng, Yingkai Wang, Yiming Zhou, Shiyan Wang, Fan Qi, Ningning Wang, Jian Ma

**Affiliations:** Faculty of Agronomy, Jilin Agricultural University, Changchun 130118, China

**Keywords:** autotetraploid rice, cuticular wax, lignin biosynthesis, phytohormone, saline-alkaline stress

## Abstract

Saline-alkali soil has posed challenges to the growth of agricultural crops, while polyploidy often show greater adaptability in diverse and extreme environments including saline-alkali stress, but its defense mechanisms in rice remain elusive. Herein, we explored the mechanisms of enhanced saline-alkali tolerance of autotetraploid rice 93-11T relative to diploid rice 93-11D, based on physiological, hormonal and transcriptomic profilings. Physiologically, the enhanced saline-alkali tolerance in 93-11T was manifested in higher soluble sugar accumulation and stronger superoxide dismutase (SOD) and peroxidase (POD) activities in leaves during 24 h after saline-alkali shock. Furthermore, various hormone levels in leaves of 93-11T altered greatly, such as the negative correlation between salicylic acid (SA) and the other four hormones changed to positive correlation due to polyploidy. Global transcriptome profiling revealed that the upregulated differentially expressed genes (DEGs) in leaves and roots of 93-11T were more abundant than that in 93-11D, and there were more DEGs in roots than in leaves under saline-alkali stress. Genes related to phytohormone signal transduction of auxin (AUX) and SA in roots, lignin biosynthesis in leaves or roots, and wax biosynthesis in leaves were obviously upregulated in 93-11T compared with 93-11D under saline-alkali condition. Collectively, 93-11T subjected to saline-alkali stress possibly possesses higher osmotic regulation ability due to cuticular wax synthesis, stronger negative regulation of reactive oxygen species (ROS) production by increasing the SA levels and maintaining relative lower levels of IAA, and higher antioxidant capacity by increasing activities of SOD and POD, as well as lignin biosynthesis. Our research provides new insights for exploring the mechanisms of saline-alkali tolerance in polyploid rice and discovering new gene targets for rice genetic improvement.

## 1. Introduction

Soil salinization is a crucial environmental constraint of the global agriculture industry and could affect 30% to 50% of the world’s arable land by 2050 due to climate change and irrational irrigation, etc. [1,2,3]. It is estimated that more than 830 million hectares of land in the world is salt-affected, half of which are saline-alkali soils [4]. In China, saline-alkali soil accounts for 25% of cultivated land, which is underutilized, mainly formed by the accumulation of sodium carbonate (Na_2_CO_3_) and sodium bicarbonate (NaHCO_3_) [5]. Saline-alkali soil is more harmful to crops than neutral saline soil (accumulation of salts such as NaCl, Na_2_SO_4_), because they not only exhibit osmotic stress, ionic toxicity and oxidative stress, but also high pH (8.5 to 11) that destroys the integrity of cell membranes, reduces root vitality and photosynthetic function, ultimately effecting plant growth and food productivity [6,7,8]. To date, most studies have focused on the molecular mechanism of plant tolerance to neutral salt [9,10,11,12,13], whereas comparatively little attention has been given to saline-alkali stress, which is increasingly becoming a serious stress factor [8,14,15,16,17,18,19]. Therefore, understanding the mechanisms of plant response to saline-alkali stress and creating novel salt-tolerant germplasm resources are necessary for the effective utilization of arable land and improving crop yields to meet the needs of world population growth [20].

Salt stress could be divided into two main phases. High salinity induces osmotic stress in the initial short term, while ion toxicity occurred due to the accumulation of phytotoxic ions in the long term, especially Na^+^ and Cl^−^, and further causes oxidative stress [21]. Plants have evolved several strategies to cope with the challenge of salinity stress, including osmoprotectants biosynthesis, activating the osmotic stress pathway, ion homeostasis regulations, mediating plant hormone signaling, synthesis of antioxidant enzyme and antioxidant compound, as well as regulating cytoskeleton dynamics and the cell wall composition [22,23]. The accumulation of various soluble osmotic adjustment substances could reduce water loss under short-term osmotic stress underlying high salinity, enhance cell expansion and stabilize cell structure under long-term osmotic stress, contributing to sustainable cell survival [24]. Evidence indicates the products of phenylpropane biosynthesis pathway have a certain effect on reactive oxygen species (ROS) scavenging [25]. This pathway results in the formation of lignin, which is one of the main components of plant cell walls and affects plant tolerance to abiotic stress [26]. Furthermore, cuticular wax also plays a role in salt tolerance, mainly through regulating residual transpiration, which might be a fundamental mechanism for plants to optimize water-use efficiency under salt stress [27]. However, most of these strategies against salt stress comprise superior performance in halophytes [28].

In general, most halophytes are dicotyledons while most important economic crops are monocotyledons. Understanding the salt-tolerance mechanism(s) of monocots will help improve the salt tolerance of cereals [29]. Rice (*Oryza sativa* L.) is a staple crop for more than half of the world’s population, and it is also an ideal model plant for genomic research in monocots [30]. Researchers have adopted several biotic approaches to develop salt-tolerant rice to facilitate better growth and stable production, including traditional breeding, molecular breeding and genetic engineering [31]. Apart from these, ploidy breeding is increasingly becoming an effective strategy to provide desired polyploid plants with improved adaptation to extreme environments [32,33]. Polyploids are traditionally classified into autopolyploids and allopolyploids, which originate from a single parent species (xx to xxxx) and two hybridizing species (xx + yy to xxyy), respectively [34]. Polyploid populations usually exhibit more resilient or selective advantages under extreme environments due to their increased genetic variation, and buffering effect of their duplicated genes [35,36,37]. These advantages are found in many tetraploid plants, such as *Arabidopsis* [38], citrange [39], watermelon [40], sour jujube [41], Anise hyssop [42], birch [43], and hexaploid plants, such as wheat [44] and spring triticale [45]. In rice, genome duplication plays a positive role on physiological and biochemical indicators of different rice varieties during seed germination and seedling growth under salt (NaCl) stress [46]. Tetraploid rice shows improved resistance to salt (NaCl) stress conferred by reducing Na^+^ uptake [47], and enhancing epigenetic regulation of jasmonic acid (JA)-related genes [48]. Upon salt (NaCl) stress, increased expression of stress-responsive genes can induce hypermethylation and inhibit transposable elements adjacent to stress-responsive genes in tetraploid rice [48]. Recently, autotetraploid rice exhibited stronger tolerance to alkaline (NaOH) stress than diploid rice, which was manifested in differences in phenotypes, physiological properties, hormone levels and gene expression patterns [49]. Accordingly, still limited research has been performed to elucidate the physiological and molecular mechanisms of polyploid rice in response to saline/saline-alkali stress.

Salt stress induces a series of changes in gene expression, protein content, and metabolite levels in rice. Genomics, transcriptomics, proteomics and metabolomics are powerful tools for identifying genes associated with salt tolerance in rice [50]. Metabolomics can be used to identify and quantify endogenous small molecule metabolites and directly reflect the metabolic status of organisms or cells at a specific time [51,52], which has become an effective way for researchers to understand the complex metabolic reactions of various abiotic pressures such as salt stress [53]. When integrated with other “omics”, metabolomics could contribute to clarifying gene functions, metabolite biosynthetic pathways, and regulatory mechanisms [54]. In recent years, integration of metabolomics and transcriptomics has been widely used to study the biosynthesis of metabolites to reveal the biosynthetic pathways of metabolites in plants [55]. Third-generation sequencing (TGS) based on single-molecular real-time (SMRT) can provide full-length transcripts information and accurate isoform annotations, which could compensate for the resolution of overall structural variations such as insertion, inversion and duplication that cannot be detected by next-generation sequencing (NGS) [56,57,58]. Therefore, this study investigated and compared the differences of physiological parameters, hormone levels and gene expressions between diploid and autotetraploid rice to explore their molecular mechanism(s) in response to saline-alkali stress. This study facilitates the exploration of the saline-alkali tolerance regulatory network of tetraploid rice and provides a basis for a comprehensive understanding of the saline-alkali tolerance mechanism of monocotyledonous crops underlying genome duplication.

## 2. Results

### 2.1. Variation of 93-11T on Plant Growth and Physiological Characteristics under Saline-Alkali Stress

**Figure 1:**
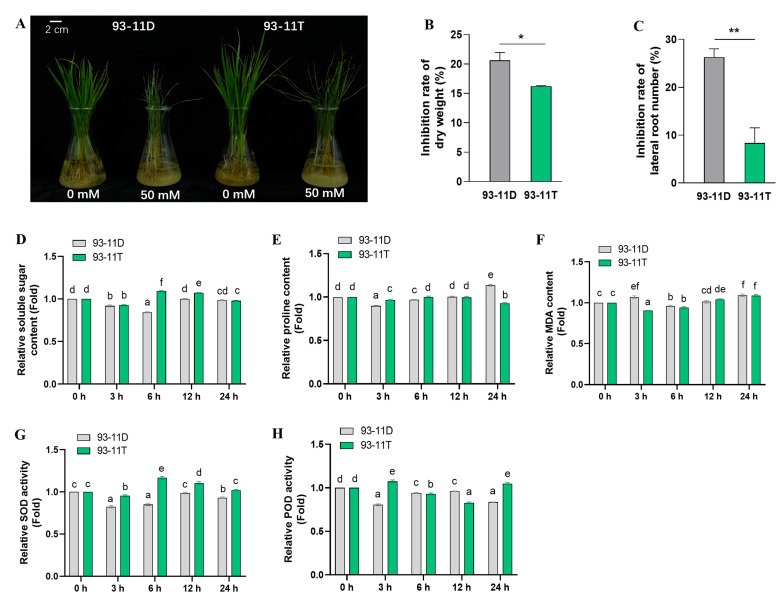
Enhanced saline-alkali tolerance in autotetraploid rice of 93-11T. (**A**) Phenotypic changes of 93-11D and 93-11T under control or 50 mM Na_2_CO_3_ treatment conditions for 3 d, scale bar = 2 cm. Inhibition rate of dry weight (**B**), and lateral root number (**C**) were measured at 7 d after stress. The samples used for measuring the relative content of soluble sugar (**D**), proline (**E**), MDA (**F**), SOD (**G**), and POD (**H**) were acquired from 93-11D and 93-11T leaves before and after Na_2_CO_3_ treatment. MDA: malondialdehyde; SOD: superoxide dismutase; POD: peroxidase. Error bar indicates SE (*n* = 3–5). Asterisks indicate significant differences between 93-11D and 93-11T with the standard of * *p* < 0.05 and ** *p* < 0.01, and different letters over bars indicate significant difference between treatments according to Duncan’s multiple range test, *p* < 0.05.

Autotetraploid 93-11T was developed from colchicine-treated 93-11D through four generations of self-pollination [59]. We investigated the phenotype of diploid and autotetraploid of 93-11 under saline-alkali stress. For convenient description, DC and TC were simplified to represent 93-11D and 93-11T under control conditions (0 h post-treatment, hpt), whereas DS and TS represent Na_2_CO_3_-treated 93-11D and 93-11T, respectively. There was no distinct difference in growth and developmental status of 93-11D and 93-11T compared with their controls at 6 hpt with 50 mM Na_2_CO_3_, whereas the leaves of 93-11D displayed less wilting at 24 hpt, and 93-11D plants exhibited more serious wilting and curling than 93-11T at 3 d post Na_2_CO_3_ treatment (Figure 1A and Supplementary Figure S1A). The dry weights of DS were reduced to a greater extent than TS (Figure 1B). In addition, the lateral root number of DS was more severely inhibited than that of TS (Figure 1C), whereas there was no significant difference in root length between of them (Supplementary Figure S1B). These parameters supported that the autotetraploid was more tolerant to saline-alkali stress than diploid progenitors.

Physiologically, the soluble sugar, as osmotic regulator, was significantly (*p* < 0.05) accumulated in TS more so than DS, at 6 hpt and 12 hpt (Figure 1D). However, the content of proline was significantly (*p* < 0.05) lower in TS than DS at 24 hpt (Figure 1E). The malondialdehyde (MDA) content, which reflects peroxidation of membrane lipid, was significantly (*p* < 0.05) lower in TS than that in DS at 3 hpt, and their levels tended to be equivalent at 6 hpt (Figure 1F). Plants have antioxidant systems to protect cells against salt stress-induced oxidative damage [60]. Therefore, we examined the activities of two antioxidant enzymes, including superoxide dismutase (SOD) and peroxidase (POD). The SOD activity of TS remained at obviously higher levels relative to DS over 24 hpt, and it was significantly (*p* < 0.05) upregulated at 6 hpt and 12 hpt in TS compared with control (Figure 1G). Interestingly, the POD activity of TS and DS exhibited diametrically opposite patterns during 24 h, and the POD activity of TS was significantly (*p* < 0.05) upregulated at 3 hpt and 24 hpt compared with control (Figure 1H). Taken together, these results suggested that genome duplication confers enhanced tolerance to saline-alkali stress in the tetraploid which displayed markedly different response to saline-alkali stress compared with the diploid.

### 2.2. Variation of 93-11T on Phytohormone Levels under Saline-Alkali Stress

**Figure 2:**
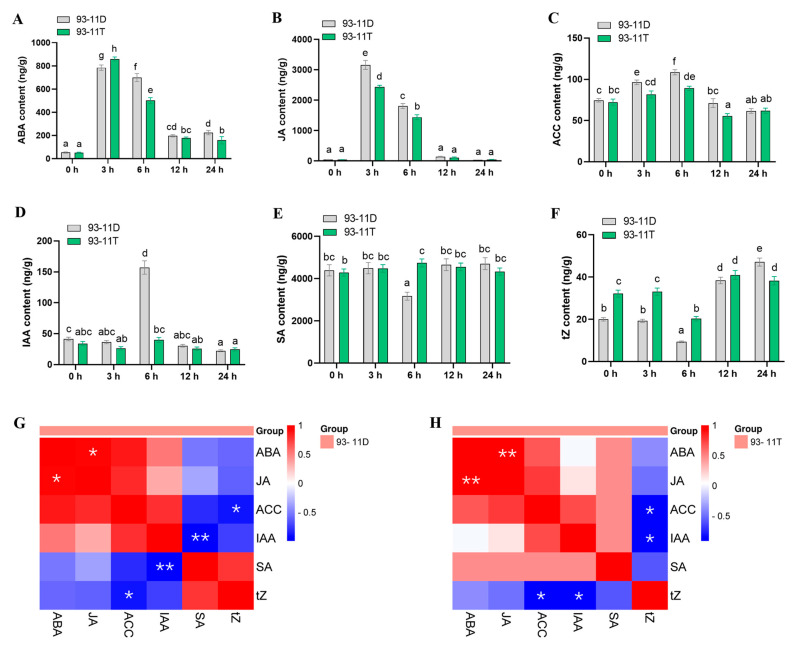
Trends and correlations of the relative changes of phytohormones in 93-11D and 93-11T before and after Na_2_CO_3_ stress. The relative contents of ABA (**A**), JA (**B**), ACC (**C**), IAA (**D**), SA (**E**), and tZ (**F**) obtained from 93-11D and 93-11T leaves before and after Na_2_CO_3_ stress at 0, 3, 6, 12 and 24 h. The correlation of each phytohormone in 93-11D (**G**), and 93-11T (**H**) were analyzed based on the Pearson correlation coefficient. Color keys and histograms show the degree of correlation. ABA: abscisic acid; JA: jasmonic acid; ACC: 1-aminocyclopropanecarboxylic acid; IAA: 3-indoleacetic acid; SA: salicylic acid; tZ: trans-Zeatin. Three biological replicates were performed for each experiment. Bars with different letters indicate significant difference between treatments according to Duncan’s multiple range test, *p* < 0.05. Asterisks indicate significant correlations between two phytohormones with the standard of * *p* < 0.05 and ** *p* < 0.01.

Phytohormones play a vital role in the signal perception and defense system mediation of plants under salt stress [22]. Hence, the content of various hormones in leaves of 93-11D and 93-11T were determined at five different time points (0, 3, 6, 12 and 24 hpt) under Na_2_CO_3_ stress. As shown in Figure 2A, abscisic acid (ABA) was induced to the maximum level at 3 hpt in both DS and TS. The content of ABA in TS was markedly higher than that in DS, and followed by a sharp decrease until 24 hpt, while the DS showed a significantly (*p* < 0.05) higher level of ABA at 6 hpt compared with TS. The JA content of DS and TS were elevated at 3 hpt and 6 hpt, and the JA content of DS was significantly (*p* < 0.05) higher than that of TS at both time points, and finally the JA content of both DS and TS decreased to the original level (Figure 2B). The content of 1-aminocyclopropanecarboxylic acid (ACC) was increased at 3 hpt and reached the maximum at 6 hpt in DS, whereas TS showed the significant (*p* < 0.05) lower level but the same trend compared with DS (Figure 2C). In case of indoleacetic acid (IAA), its content in DS was significantly (*p* < 0.05) increased with a higher level than that in TS at 6 hpt, whereas the IAA content of TS remained at lower levels within 24 h (Figure 2D). Interestingly, the salicylic acid (SA) content of DS showed an opposite trend to that of IAA at 6 hpt, while the SA content of TS was significantly (*p* < 0.05) increased compared with control and higher than DS at 6 hpt (Figure 2E). Unlike other hormones, the initial content of trans-Zeatin (tZ) in TS was obviously higher than that in DS, followed by a significant (*p* < 0.05) decrease at 6 hpt, and then a rapid increase at 12 hpt and 24 hpt, and finally the content of tZ was significantly (*p* < 0.05) higher in DS than that in TS at 24 hpt (Figure 2F).

Crosstalk among various phytohormone signaling regulates the balance between plant growth and defense, which are the core of plant stress response [61,62]. Accordingly, hormone correlation studies were performed throughout the whole stress process. In DS, hormones of ABA, JA, ACC and IAA were positively correlated with each other, and SA was positively correlated with tZ, while both of them showed negative correction with other hormones during 24 h (Figure 2G). By contrast, SA was negatively correlated with tZ and positively correlated with other hormones in TS (Figure 2H). Furthermore, IAA was negatively correlated with SA in DS at a significant (*p* < 0.01) level, whereas it was negatively correlated with tZ in TS at a significant (*p* < 0.05) level (Figure 2G,H). These results suggested that the synergistic and antagonistic interactions among various hormones in leaves of diploid and tetraploid were different in response to saline-alkali stress, and SA or IAA might play an important role in the positive response of TS.

### 2.3. Comparison of Transcriptional Profiling between 93-11D and 93-11T in Response to Saline-Alkali Stress

To explore the molecular differences underlying saline-alkali tolerance between DS and TS, the transcriptional profiles were analyzed by NGS combined with TGS. For NGS, 24 cDNA libraries were separately sequenced by an Illumina high-throughput NGS platform. After removing the low-quality reads and possible contaminations, a total of ~198.5 Gb clean data with Q30 > 88.58% and GC percentage between 52.01% and 61.44% were used for further analysis (Supplementary Table S1). For TGS, leaf and root samples of 93-11D and 93-11T were mixed separately to produce two libraries and sequenced on the PacBio RS II platform; a total of ~31.45 Gb subread bases and 812,249 reads of insert (ROI) were generated. After that, the full-length reads were classified according to the presence of barcoded primers and poly (A) tails, and 354,524 full-length non-chimeric (FLNC) reads were identified. On average, 43.82% of all ROIs were full-length reads, and the artificial concatemers displayed 0.38% of the full-length reads. The length distribution of FLNC was consistent with the cDNA size distribution. After clustering redundant sequences, 152,538 consensus isoforms, including 125,447 high-quality isoforms, were obtained (Supplementary Table S2).

**Figure 3:**
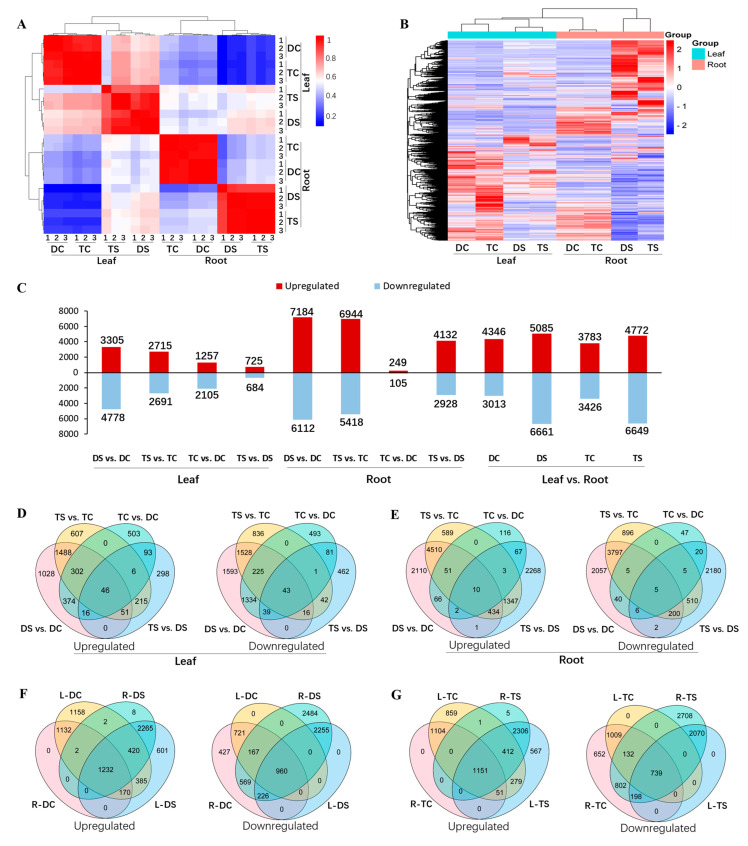
Analysis data of next-generation sequencing (NGS) and third-generation sequencing (TGS)-derived DEGs in 93-11D and 93-11T with or without Na_2_CO_3_ treatment. (**A**) Hierarchical clustering of 24 samples according to the correlation coefficient between each sample. (**B**) Heat map hierarchical clustering of the overall gene expression. (**C**) Statistics of up- (in red) and down-regulated (in blue) DEGs in leaf, root and leaf vs. root for each pairwise comparison. Venn diagrams of up- and down-regulated DEGs in leaves (**D**), roots (**E**), leaf/root of diploid (**F**), and leaf/root of tetraploid (**G**). DC and TC represent diploid and tetraploid without stress, respectively; DS and TS represent diploid and tetraploid under Na_2_CO_3_ stress, respectively.

We next performed hierarchical clustering analysis on the test samples to estimate the expression values of all genes. As shown in Figure 3A, leaf and root samples were clustered separately in the first clade, and the three replicates of each treatment were mostly clustered together, indicating that gene expression profiles of the samples used in this study were highly consistent. A heatmap was generated for all differentially expressed genes (DEGs) to investigate the transcriptomes of DS and TS, which showed that the gene expression patterns changed markedly between DS and TS (Figure 3B). To determine the differentially expressed salt-responsive genes in the leaves and roots of 93-11D and 93-11T, we compared the expression values in pairs, (I) leaf or root: DS vs. DC, TS vs. TC, TC vs. DC, and TS vs. DS (Supplementary Tables S3 and S4); (II) leaf (L) vs. root (R): L-DC vs. R-DC, L-DS vs. R-DS, L-TC vs. R-TC and L-TS vs. R-TS (Supplementary Table S5). The results showed that a total of 8083 differentially expressed genes (DEGs, 3305 upregulated) were obtained in DS vs. DC, while only 5406 DEGs (2715 upregulated) were identified in TS vs. TC in leaves (Figure 3C,D). However, we found more upregulated genes in TS than DS in leaves (Figure 3C). In roots, there were 13,296 DEGs (7184 upregulated) in DS vs. DC, as well as 12,362 DEGs (6944 upregulated) in TS vs. TC (Figure 3C,E). Likewise, more genes were upregulated in TS than DS (Figure 3C). Notably, the total number of DEGs in roots were higher than that in leaves, in response to Na_2_CO_3_ stress (Figure 3C–E). In leaves, compared with roots, there were more upregulated genes in DC (leaf: 4346 vs. root: 3013) and TC (leaf: 3783 vs. root: 3426), whereas more downregulated genes in DS (root: 6661 vs. leaf: 5085) and TS (root: 6649 vs. leaf: 4772) (Figure 3C,F,G). To assess the reliability of RNA seq data, the expression patterns of eight randomly selected genes were analyzed using quantitative real-time polymerase chain reaction (qRT-PCR). There was a strong positive correlation (*R*^2^ = 0.8842) between RNA seq data and qRT-PCR results, implying the reliability of RNA seq data (Supplementary Figure S2). Taken together, these results suggested that roots are more sensitive than leaves under saline-alkali stress, and the autotetraploid behaves more actively than diploid in response to saline-alkali stress at the transcriptional level.

### 2.4. Functional and Pathway Enrichment Analysis of Trend Genes between 93-11D and 93-11T

**Figure 4:**
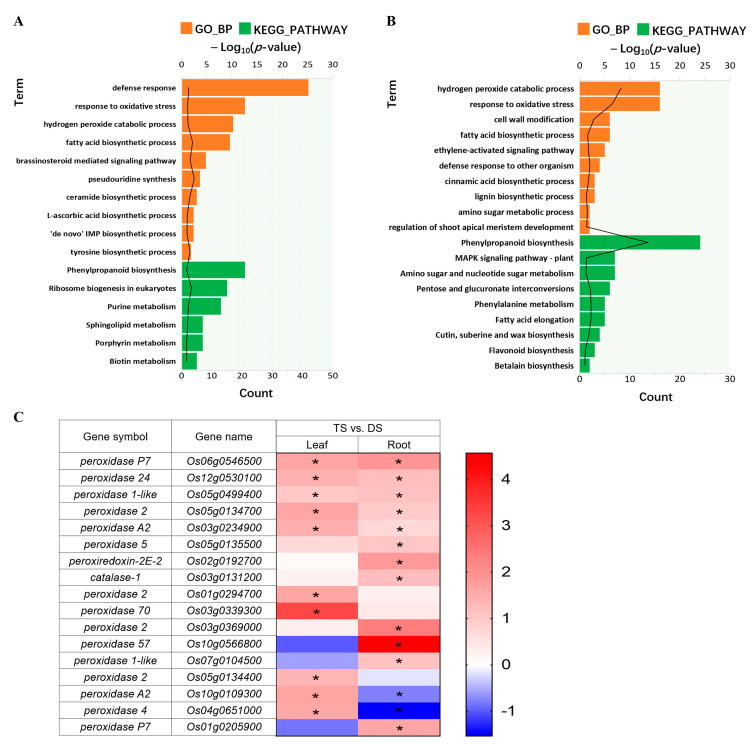
GO functional and KEGG enrichment analysis for trend DEGs in 93-11D and 93-11T under Na_2_CO_3_ stress (TS vs. DS). Uptrend genes in roots (**A**), and leaves (**B**). The black line represents −log10 (*p*-value), refer to the upper scale; the number of enriched genes refer to the bottom scale; orange column represents GO_BP, green column represents KEGG pathway. GO: gene ontology; KEGG: Kyoto Encyclopedia of Genes and Genomes; BP: biological processes; DEGs: differentially expressed genes. (**C**) Heatmap of DEGs involved in peroxidase superfamily in leaves and roots of 93-11T relative to 93-11D (TS vs. DS). DS and TS represent diploid and tetraploid under Na_2_CO_3_ stress, respectively. The scale bar shows the value of log_2_ fold change (FC) with * *p* < 0.05.

To further understand the underlying mechanism(s) of tetraploid rice to saline-alkali stress, we explored the biological classification and Kyoto Encyclopedia of Genes and Genomes (KEGG) pathway enrichment analysis of DEGs by comparing the transcriptomes of TS and DS (TS vs. DS, Supplementary Tables S3 and S4). Several gene ontology (GO) terms in biological processes (BP) were commonly upregulated in roots and leaves, including ‘response to oxidative stress’, ‘hydrogen peroxide catabolic process’ and ‘fatty acid biosynthetic process’ (Figure 4A,B). Other terms markedly upregulated in roots were ‘defense response’, ‘brassinosteroid mediated signaling pathway’, ‘pseudouridine synthesis’, ‘ceramide biosynthetic process’ and ‘tyrosine biosynthetic process’ (Figure 4A). While in leaves, they were ‘cell wall modification’, ‘ethylene-activated signaling pathway’, ‘cinnamic acid biosynthetic process’, ‘lignin biosynthetic process’, ‘amino sugar metabolic process’ and ‘regulation of shoot apical meristem development’ (Figure 4B). Correspondingly, the downtrend DEGs in roots were markedly enriched in ‘protein glycosylation’, ‘glutathione metabolic process’, ‘aromatic compound biosynthetic process’ and ‘tryptophan biosynthetic process’ (Supplementary Figure S3A). In leaves, they were ‘response to water deprivation’, ‘response to abscisic acid’, ‘response to salt stress’, ‘response to hydrogen peroxide’ and ‘phosphatidylcholine biosynthetic process’ (Supplementary Figure S3B).

The enriched KEGG pathways of uptrend DEGs in roots were abundant in ‘phenylpropanoid biosynthesis’, ‘ribosome biogenesis in eukaryotes’ and ‘purine metabolism’ (Figure 4A). In leaves, they were ‘phenylpropanoid biosynthesis’, ‘MAPK signaling pathway-plant’, ‘pentose and glucuronate interconversions’, ‘phenylalanine metabolism’, ‘fatty acid elongation’ and ‘cutin, suberine and wax biosynthesis’ (Figure 4B). The KEGG pathways of downtrend DEGs in roots and leaves were highly enriched in ‘plant hormone signal transduction’, ‘MAPK signaling pathway-plant’, ‘glutathione metabolism’ and ‘phenylalanine, tyrosine and tryptophan biosynthesis’ (Supplementary Figure S3A), and ‘galactose metabolism’, respectively (Supplementary Figure S3B).

Notably, most of the DEGs contained in the BP of ‘response to oxidative stress’ and ‘hydrogen peroxide catabolic process’, co-enriched in roots and leaves, belong to the peroxidase superfamily. Compared with DS, ten and twelve DEGs were significantly (*p* < 0.05) upregulated in leaves and roots of TS, respectively, and two genes were significantly (*p* < 0.05) downregulated in roots (Figure 4C and Supplementary Table S6). This result implied peroxidase family members probably provide powerful support for the scavenging of ROS caused by saline-alkali stress in autotetraploid rice.

In addition, KEGG enrichment analysis showed that 21 DEGs related to ‘plant hormone signal transduction’ were significantly (*p* < 0.05) upregulated in DS (Supplementary Figure S3A). Thus, we examined the functional annotations of 21 genes and found that five genes belonged to the auxin signal transduction pathway, one gene belonged to the ethylene biosynthesis pathway, one gene was jasmonic acid-amido synthetase, and the rest belonged to transcription factors (TFs) or proteins (Supplementary Table S7). Among them, two genes annotated with ‘auxin-responsive protein SAUR36′ and one gene annotated with ‘ethylene insensitive 3-like 3 protein’ were associated with senescence, according to previous studies [63,64]. Evidences have shown that avoiding or delaying plant senescence is an important strategy to improve plant salt tolerance [65,66]. Therefore, this result suggested that the sensitivity of diploid rice to saline-alkali stress may be associated with the upregulation of senescence-related genes.

### 2.5. Transcriptomic Analysis of Lignin Biosynthesis Pathway in 93-11T under Saline-Alkali Stress

**Figure 5:**
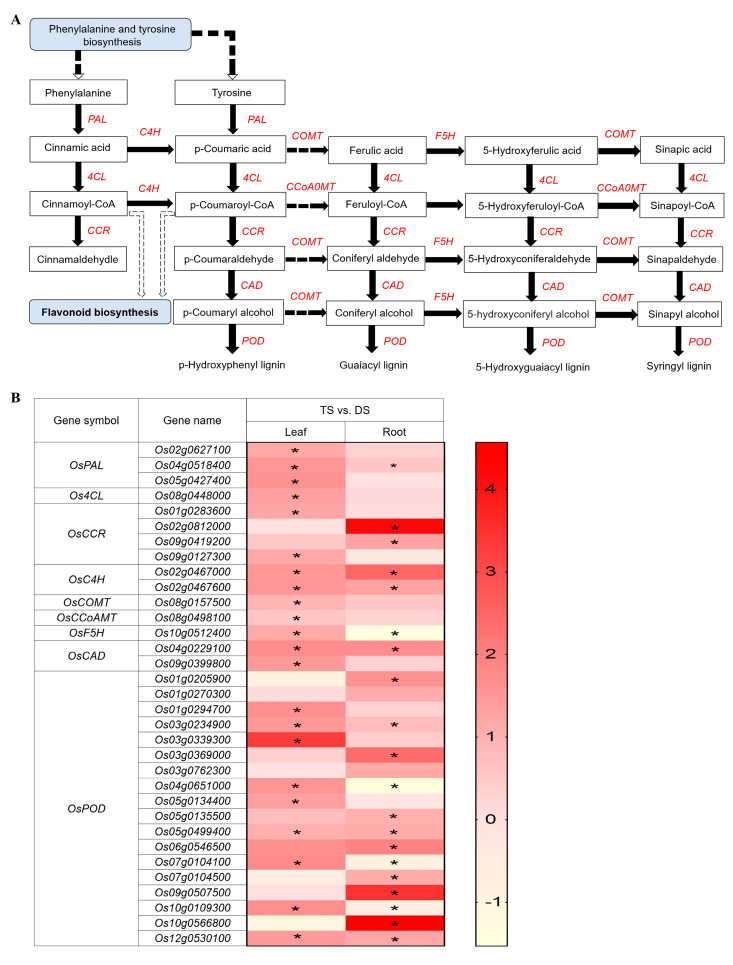
Schematic view of genes involved in the lignin biosynthesis pathway and heatmap analysis of DEGs under Na_2_CO_3_ stress (TS vs. DS). (**A**) Overview of lignin biosynthesis pathway. The solid lines indicate direct interactions, and the dashed lines indicate indirect interactions. The arrows indicate stimulatory effects, whereas the T sharp symbol indicates inhibitory effects. (**B**) Heatmap analysis of DEGs enriched in the lignin biosynthesis pathway in leaves and roots. PAL, phenylalanine ammonia-lyase; 4CL, 4-coumarate-CoA ligase; CCR, cinnamoyl CoA reductase; C4H, trans-cinnamate 4-monooxygenase; COMT, caffeic acid 3-O-methyltransferase; CCoAMT, caffeoyl-CoA O-methyltransferase; F5H, ferulate-5-hydroxylase; CAD, cinnamyl alcohol dehydrogenase; POD, peroxidase. DS and TS represent diploid and tetraploid under Na_2_CO_3_ stress, respectively. Expression scores are shown as log_2_ fold change (FC) with * *p* < 0.05.

To verify metabolic pathway(s) potentially associated with the enhanced salt tolerance in 93-11T, we combined the information of GO_BP and KEGG pathways and verified that the DEGs were highly enriched in the phenylpropanoid biosynthesis (ko00940) involved in lignin biosynthesis in leaves or roots of 93-11T compared with 93-11D (TS vs. DS). In the lignin biosynthesis pathway, 9 families covering 33 upregulated DEGs were identified in leaves or roots of TS, including phenylalanine ammonia-lyase (PAL), 4-coumarate–CoA ligase (4CL), cinnamoyl-CoA reductase (CCR), trans-cinnamate 4-monooxygenase (C4H), caffeic acid 3-O-methyltransferase (COMT), caffeoyl-CoA O-methyltransferase (CCoAMT), ferulate-5-hydroxylase (F5H), cinnamyl alcohol dehydrogenase (CAD), and peroxidase (POD) (Figure 5A). Among them, genes contained in the 4CL, COMT, CCoAMT and F5H families were significantly (*p* < 0.05) upregulated in leaves only, while the genes contained in other families were significantly (*p* < 0.05) upregulated in both leaves and roots of TS, and the number of significantly upregulated genes was higher in leaves (Figure 5B and Supplementary Table S8). In the lignin biosynthesis pathway, most of the identified DEGs belonged to the OsPOD family and were obviously upregulated in both leaves and roots of TS (Figure 5B). These results suggested that the lignin biosynthesis pathway might be one of the main strategies of autotetraploid rice different from diploid to cope with saline-alkali stress.

### 2.6. Transcriptomic Analysis of Wax Biosynthesis Pathway in 93-11T under Saline-Alkali Stress

**Figure 6:**
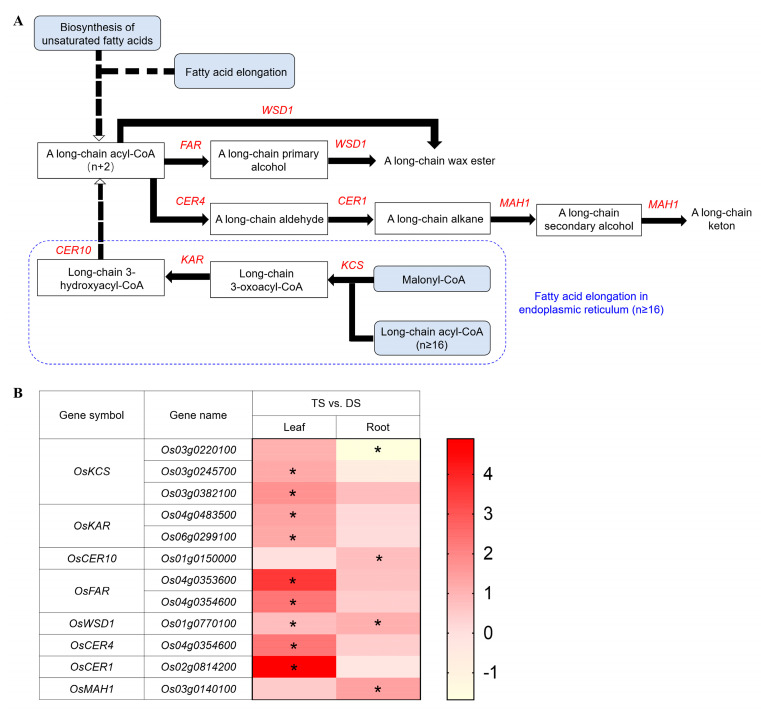
Schematic view of genes involved in the wax biosynthesis pathway and heatmap analysis of DEGs under Na_2_CO_3_ stress (TS vs. DS). (**A**) Overview of wax biosynthesis pathway and fatty acid elongation process (in blue dashed box). The solid lines indicate direct interactions, and the dashed lines indicate indirect interactions. The arrows indicate stimulatory effects, whereas the T sharp symbol indicates inhibitory effects. (**B**) Heatmap analysis of DEGs enriched in the wax biosynthesis pathway in leaves and roots. KCS: 3-ketoacyl-CoA synthase; KAR: very-long-chain 3-oxoacyl-CoA; OCE10: very-long-chain enoyl-CoA reductase; FAR: alcohol-forming fatty acyl-CoA reductase; WSD1: wax-ester synthase/diacylglycerol O-acyltransferase; CER4: acyl-CoA reductase; CER1: aldehyde decarbonylase; MAH1: midchain alkane hydroxylase. DS and TS represent diploid and tetraploid under Na_2_CO_3_ stress, respectively. Expression scores are shown as log_2_ fold change (FC) with * *p* < 0.05.

Plant cuticular wax plays an important role in salt tolerance by regulating residual transpiration to improve water use efficiency under hyperosmotic conditions [67,68]. The transcriptomic analysis showed that six DEGs from three families, including 3-ketoacyl-CoA synthase (KCS), very-long-chain 3-oxoacyl-CoA (KAR) and very-long-chain enoyl-CoA reductase (OCE10), were found in fatty acid elongation process (Figure 6A), which are the basis of wax biosynthesis. Furthermore, six DEGs from five families, including alcohol-forming fatty acyl-CoA reductase (FAR), wax-ester synthase/diacylglycerol O-acyltransferase (WSD1), acyl-CoA reductase (CER4), aldehyde decarbonylase (CER1) and midchain alkane hydroxylase (MAH1), were obtained in the wax biosynthesis pathway (Figure 6A). Eight DEGs from KCS, KAR, FAR, CER4 and CER1 families were obviously upregulated in leaves of TS only, and two DEGs from CER10 and MAH1 families were significantly (*p* < 0.05) upregulated in roots only (Figure 6B and Supplementary Table S9). In this pathway, almost all genes were significantly (*p* < 0.05) upregulated in leaves. These data suggested the cuticle wax formation process may increase the osmotic regulation ability by regulating residual transpiration in tetraploid leaves which probably confer a better way to deal with saline-alkali stress than diploid.

### 2.7. Variation of Phytohormone Regulations in 93-11T in Response to Saline-Alkali Stress

**Figure 7:**
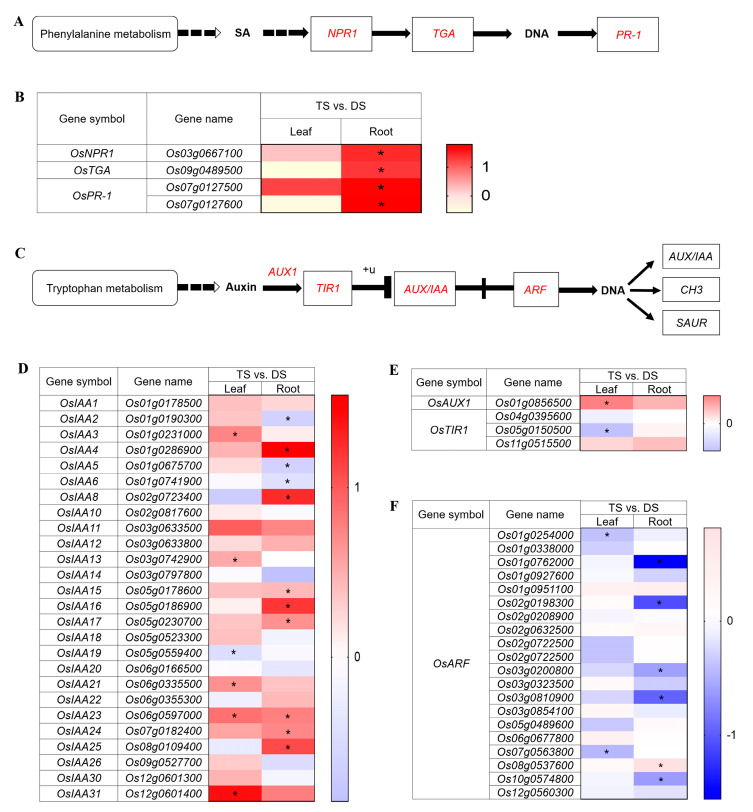
Schematic view of genes involved in the SA and auxin signaling pathway and heatmap analysis of DEGs under Na_2_CO_3_ stress (TS vs. DS). (**A**) Overview of SA signaling pathway. The solid lines indicate direct interactions, and the dashed lines indicate indirect interactions. The arrows indicate stimulatory effects, whereas the T sharp symbol indicates inhibitory effects. (**B**) Heatmap analysis of DEGs enriched in the SA signaling pathway in leaves and roots. (**C**) Overview of auxin signaling pathway. (**D**–**F**) Heatmap of auxin signaling pathway related gene expression in leaves and roots. +u means ubiquitination. SA: salicylic acid; NPR1: regulatory protein NPR1; TGA: transcription factor TGA; PR-1: pathogenesis-related protein 1; AUX1: auxin influx carrier; TIR1: transport inhibitor response 1; AUX/IAA: auxin-responsive protein IAA; ARF: auxin response factor; GH3: auxin-responsive GH3 gene family; SAUR: SAUR family protein. DS and TS represent diploid and tetraploid under Na_2_CO_3_ stress, respectively. Expression scores are shown as log_2_ fold change (FC) with * *p* < 0.05.

Phytohormones play an important role in plant growth and serve signals in regulating plant adaptation to various environmental pressures, such as salt stress [67]. Therefore, we further analyzed the DEGs annotated in signal transduction and biosynthesis pathways of phytohormones. Evidence indicated that the regulatory protein non-expressor of pathogenesis-related genes 1 (NPR1)-dependent SA signaling plays a central role in salt and oxidative stress tolerance in *Arabidopsis* [7]. By analyzing the DEGs related to the SA signal transduction pathway, we found one gene of *NPR1*, one gene TF of TGA and two genes of pathogenesis-related protein 1 (*PR-1*) were significantly (*p* < 0.05) upregulated in roots of TS rather than leaves, compared with DS (Figure 7A,B and Supplementary Table S10). This result may imply that the tetraploid root plays an important role in saline-alkali tolerance through the NPR1-dependent SA signaling pathway. Furthermore, in the auxin signal transduction pathway (Figure 7C), *AUX/IAA* as a negative regulator of auxin (AUX) signaling, contributes to tolerance to abiotic stress in *Arabidopsis* [69]. In addition, *Arabidopsis* slows plant growth to improve salt tolerance by maintaining a low-signal response of AUX receptor genes, transport inhibitor response 1 (*TIR1*) and auxin signaling F-box 2 (*AFB2*) [70,71]. All DEGs associated with *AUX/IAA* in leaves and roots of TS were investigated relative to DS. The results showed 73% of *OsIAA* genes in leaves were upregulated, five of which were significantly (*p* < 0.05) upregulated. Simultaneously, 58% of *OsIAA* genes were upregulated in roots, eight of which were significantly (*p* < 0.05) upregulated (Figure 7D and Supplementary Table S10). Furthermore, auxin influx carrier *OsAUX1* gene was upregulated in both leaves and roots of TS, and two genes (*Os04g0395600* and *Os05g0150500*) of transport inhibitor response 1 (*OsTIR1*) were downregulated in leaves of TS, while another *OsTIR1* gene (*Os11g0515500*) was upregulated in both leaves and roots of TS (Figure 7E and Supplementary Table S10). Moreover, the auxin response factors (*ARF*) family plays a key role in regulating the expression of AUX response genes [72]. Most of the DEGs related to *OsARF* were downregulated in leaves and roots of TS, including two significantly (*p* < 0.05) downregulated in leaves and five significantly (*p* < 0.05) downregulated in roots (Figure 7F and Supplementary Table S10). Taken together, these results suggested that the distinct SA and IAA signal transduction relative to diploid may play a positive role in the tolerance of autotetraploid to saline-alkali stress.

## 3. Discussion

**Figure 8:**
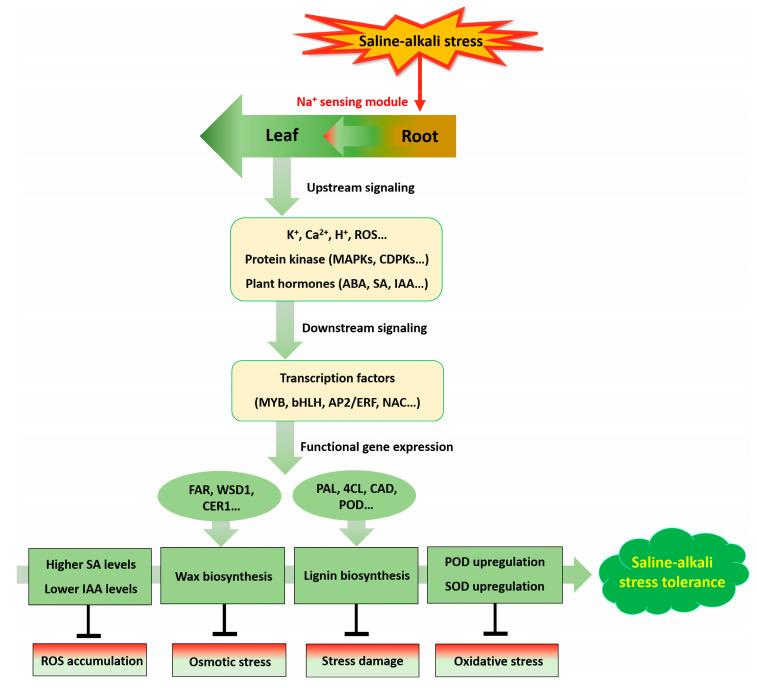
A model of signal transduction and regulation of secondary metabolism in autotetraploid rice against saline-alkali stress. The arrows indicate stimulatory effects, and the T sharp symbol indicates inhibitory effects.

Saline-alkali stress manifests not only in high salinity, but also inordinately high alkalinity (high pH), which severely impairs the growth of rice seedlings by causing serious root cell injury and death, eventually leading to the withering and even death of the whole plant [8]. Nevertheless, whole genome duplication offers the possibility of genotypic and phenotypic changes and improves the plasticity of polyploids to adapt to extreme environments [35,73]. This is consistent with our investigation that autotetraploid rice was phenotypically more tolerant to high alkalinity (pH = 11.39) than diploid progenitors (Figure 1A–C). However, the mechanisms by which polyploids enhance adaptation and fitness in saline-alkali stress are poorly understood. Our study identified and provided new insights of superior saline-alkali tolerance strategies in autotetraploid rice compared to diploid, based on comprehensive metabolomic and transcriptomic profilings (Figure 8). To adapt and survive under high salinity conditions, upstream signaling responses are triggered after early perception of excessive Na^+^, such as K^+^, Ca^2+^, H^+^, various protein kinases (MAPKs, CDPKs, etc.), phospholipid, ROS, and plant hormones are involved in the complex signal transduction network. Subsequently, TFs that regulate stress-responsive genes are induced and result in high-efficiency expression of functional genes such as ion transporter genes and antioxidant genes (Figure 8). This process has important implications for plants to develop resistance [74].

Rice roots in direct contact with saline solution have to withstand osmotic stress and ionic stress under saline conditions [75]. With the persistence of extra-root high-salt stress, osmotic stress occurs immediately in plants, thereby inhibiting plant water uptake, cell expansion, and lateral bud development. On the other hand, salt-induced oxidative stress may damage the structure of the cell membrane, as excess production of ROS triggers lipid and protein peroxidation [47]. However, SOD is a major antioxidant that defends against cell damage by converting O_2_^•−^ into H_2_O_2_, and POD can remove H_2_O_2_ in plants and prevent the cell membrane from oxidation by H_2_O_2_ [76]. In this study, soluble sugar was accumulated in leaves of tetraploid rice in the early 6–12 h to cope with high saline-alkali stress, while proline accumulation was found in leaves of diploid rice at 24 h (Figure 1D,E). The relative MDA content of diploid and tetraploid was increased, caused by excessive ROS (Figure 1F). Nevertheless, POD was first activated within 3 h in leaves of tetraploid but not in diploid under saline-alkali stress, and SOD was raised when POD start to decrease; finally POD was activated again to catalyze H_2_O_2_ produced by SOD metabolism (Figure 1G,H). This process showed a distinct antioxidant pattern in autotetraploid compared with diploid, which could subsequently rescue the elevated MDA content in tetraploid caused by lipid membrane peroxidation over a period of time.

In rice, ethylene may regulate the accumulation of ROS by modulating auxin biosynthesis and signal transduction, thereby affecting its salinity tolerance [77]. Evidence indicated exogenous application of auxin analogue naphthalene acetic acid (NAA) can induce ROS production, while the inhibition of auxin biosynthesis by aminoethoxyvinylglycine (AVG) suppressed ROS production [78]. In the current study, leaves of tetraploid rice maintained an obviously lower level of IAA in leaves than diploid at 6 hpt (Figure 2D and Figure 7C–F). Thus, we speculated that ACC as the precursor of ethylene, may prevent the excessive accumulation of ROS by limiting auxin biosynthesis, thereby enhancing the saline-alkali tolerance of tetraploid. Upon salt stress, the increase of endogenous SA levels could lead to a significant decrease of ROS and Na^+^ accumulation across the plant, whereas SA-deficient plants produced elevated levels of superoxide and H_2_O_2_ [79]. Herein, we found that SA content in leaves of tetraploid was markedly elevated in contrast to that in diploid at 6 hpt, which indicates that SA may have a positive regulatory effect on leaves of tetraploid in response to saline-alkali stress by negatively regulating ROS production and/or Na^+^ accumulation. Additionally, correlation studies revealed a positive correlation between SA and IAA in leaves of tetraploid in response to saline-alkali stress (Figure 2H), which could help maintain the extensibility of leaf cells to promote better plant growth under stress condition according to earlier reports [80,81].

Previous studies revealed that cytokinins (CKs) functionally regulate plant adaptation to environmental stresses. For instance, exogenous application of CKs improved the salt tolerance of *Solanum melongena* [82]. In rice, *cytokinin oxidase 2* (*OsCKX2*) encodes an enzyme that degrades CK, and knockdown of this enzyme results in better vegetative growth, higher relative water content, and photosynthetic efficiency than their wild-type under salt stress [83]. Our study showed the tZ content of diploid and tetraploid was negatively regulated at 6 hpt, and maintained a significantly high level in leaves of tetraploid; whereas the tZ content of both diploid and tetraploid increased rapidly from 12 h, and the tZ content of diploid was significantly higher than that of 93-11T at 24 hpt (Figure 2F). In *Arabidopsis*, the reduction of CK levels can improve the survival rate of CK-deficient plants by minimizing water loss and maintaining the intact membrane structure, as well as conferring greater salt tolerance in CK-deficient plants [84]. Therefore, we suspected that genome duplication allows leaves of tetraploid to contain higher tZ content than the diploid ancestor, which may confer higher viability in tetraploid rice to resist short-term saline-alkali stress, while for long-term saline-alkali stress, it is necessary to reduce the CK content appropriately to avoid water loss and membrane damage to achieve the ultimate goal of salt tolerance.

Generally, plant cell walls undergo lignification under stress [85]. Lignin, as a major component of plant cell walls, enhances plant resistance to biotic and abiotic stresses [86]. The transcriptomic analysis of our study illustrated that the expression of 33 upregulated DEGs involved in phenylpropanoid biosynthesis and related to the lignin biosynthetic pathway were highly influenced by saline-alkali shock in leaves or roots of tetraploid rice (Figure 5). This indicated tetraploid rice may resist saline-alkali stress by solidifying cell wall through the lignin biosynthesis pathway. In addition, the genes involved in lignin biosynthesis are largely regulated by lignin-specific TFs at the transcriptional level [87]. In *Eucalyptus*, the R2R3 MYB family of *EgMYB2* regulates two lignin biosynthetic genes, *CCR* and *CAD* [88]. The *Arabidopsis AtMYB61* has been shown to function as a master regulator for secondary cell-wall development [89]. In rice, bHLH TF of *OsbHLH034* plays a positive regulatory role in JA-mediated defense response by elevating lignin content [90]. In *Eriobotrea japonica*, AP2/ERF stimulated lignification of fruits by interaction with MYB TFs [91]. In addition, AP2/ERF TF positively regulates lignan biosynthesis in *Isatis indigotica* by activating SA signaling and genes involved in lignan/lignin pathway [92]. Upon drought and salt stress, *AgNAC1*, a TF of celery NAC, was overexpressed in *Arabidopsis*, which improved the drought and salt tolerance of *Arabidopsis* due to the increased of SOD and POD activities and lignin content [86]. Our transcriptomic analysis demonstrated that seven MYB genes, including *OsMYB2* and *OsMYB61*, six bHLH genes and six AP2/ERF genes was markedly upregulated in tetraploid leaves upon saline-alkali stress. Thus, we suspect that lignin biosynthesis pathway-related genes might be regulated by MYB, bHLH, AP2/ERF, and/or other TFs to achieve their functions in tetraploid rice (Figure 8).

Cuticle wax is the outermost hydrophobic layer of aerial plant tissues, which plays a vital role in protecting plants from external environmental stresses, and serves as a barrier to excessive non-stomatal transpiration [93]. In *Arabidopsis*, *MYB94* and *MYB96* concertedly activate wax biosynthesis by regulating the expression of relative genes such as *FAR3*, *WSD1*, *KCS2*/*DAISY*, *CER2*, *ECR*, *KCS1*, *KCS6* and *KCR1*, confers drought tolerance in plants [94,95]. The *WRINKLED4 (WRI4)* encoding an ethylene-responsive factor AP2/ERF, is involved in activating cuticular wax biosynthesis in *Arabidopsis* stems and serves as a transcriptional activator to regulate the expression of *WSD1*, *KCR1*, *LACS1*, *PAS2* and *ECR* [96]. The TFs of MYB and AP2/ERF obtained in this study may be responsible for regulating the genes expression related to the cuticular wax biosynthesis in leaves of tetraploid rice in response to saline-alkali stress.

In summary, the defense process of autotetraploid 93-11T in response to saline-alkali stress may be the accumulation of MDA, ROS, soluble sugar and other substances in a short period, and the signal transduction activates the expression of TFs such as MYB, bHLH, AP2/ERF. By promoting antioxidant enzyme activities, as well as regulating the expression of genes related to phytohormones (*OsNPR1*, *OsTGA*, *OsPR-1*; *OsIAA4*, *OsIAA8*, *OsIAA16* and *OsIAA25*) in leaves or roots, lignin biosynthesis (*OsPAL*, *Os4CL*, *OsCCR*, *OsC4H*, *OsCOMT*, *OsCCoAMT*, *OsF5H*, *OsCAD* and *OsPOD*) in leaves or roots, and wax biosynthesis (*OsFAR*, *OsWSD1*, *OsCER4*, *OsCER1* and *OsMAH1*) in leaves, indicate that avoiding ionic toxicity and oxidative damage, as well as osmotic adjustment, are the key mechanisms of autotetraploid rice 93-11T, superior to its diploid ancestor against high saline-alkali shock. In particular, we propose a hypothesis that activation of lignin and wax biosynthetic pathways is involved in the resistance of tetraploid rice to Na_2_CO_3_, which will provide new clues to reveal the molecular mechanisms of saline-alkali tolerance in polyploid rice. Further work is necessary to verify these new findings with more targeted experiments.

## 4. Materials and Methods

### 4.1. Plant Materials, Growth Conditions and Stress Treatments

Diploid (93-11D) and autotetraploid (93-11T) of *Oryza sativa* L. ssp. *indica* ‘Yangdao 6′ cultivar 93-11 were used in this study. The 93-11T was developed from tissue culture of 93-11D and artificially synthesized by colchicine treatment [59], and self-pollinated for four generations to ensure genetic stability. Chromosomes were counted as previously described [49]. Seeds of 93-11D and 93-11T were sterilized by 10% sodium hypochlorite (NaOCl) for 15 min and rinsed three times with distilled water, then soaked in tap water for 1 d at 35 °C in the dark. The well-germinated seeds were transferred onto moist tissue paper in petri dish until diphyllous stage, then cultured in nutrient solution [97]. Seedlings at the trefoil stage were exposed to high saline-alkali stress (50 mM Na_2_CO_3_ in nutrient solution, pH = 11.39) for 1 to 7 d. This hydroponic experiment was carried out in a growth chamber maintained at 28/24 °C (16 h light period/8 h dark period) in the college of agronomy of Jilin Agricultural University (Changchun, China). Fifteen representative plants with vigorous growth were selected for stress treatment in each replicate group. Samples (leaves or roots) were collected for physiological analysis, phytohormone profiling, NGS and TGS or immediately frozen in liquid nitrogen and stored at −80 °C for further analysis. Each sample per analysis had at least three biological replicates.

To evaluate the saline-alkali tolerance of 93-11D and 93-11T, the plant dry weight, root length and lateral root number were determined at 7 d post treatment with 50 mM Na_2_CO_3_. Dry weight measurements were performed after the samples were dried at 80 °C for 3 d. Inhibition rate was estimated by the ratio of the difference between the control and the treated sample to the control sample. The data were calculated from five biological replicates.

### 4.2. Physiological Measurements

The measurements of soluble sugar, proline and MDA content, as well as SOD and POD activities in leaves (93-11D and 93-11T) at 0, 3, 6, 12 and 24 hpt were conducted as previously described [49]. Briefly, (i) soluble sugar content: 5 mL of anthrone reagent were added to 1 mL of extract, incubated at 95 °C for 15 min. After cooling to room temperature, absorbance was measured at 625 nm. (ii) Proline content: The leaf samples were homogenized in 10 mL of 3% aqueous sulfosalicylic acid and filtered by filter paper. Two milliliters of filtrate was reacted with 2 mL of acidnihdrin and 2 mL of glacial acetic acid at 100 °C for 1 h, then placed in an ice bath. The reaction mixture was extracted with 4 mL of toluene, the chromophore containing toluene was aspirated from the aqueous phase. The absorbance of the solution was measured at 520 nm [98]. (iii) MDA content: The leaf samples were homogenized in 5 mL of 10% trichloroacetic acid (TCA), then centrifuged at 10,000× *g* for 10 min, the supernatant was added to 0.6% thiobarbituric acid in 10% TCA, and incubated in a water bath at 95 °C for 30 min, the reaction was stopped with ice. The absorbance of the solution was measured at 450, 532, and 600 nm. MDA contents (nmol/g fresh weight) were then calculated by the following formula: [6.45 × (A_532_–A_600_) − 0.56 × A_450_]/fresh weight. (iv) SOD and POD activities: 500 mg of triturated samples were mixed with 50 mM potassium phosphate buffer (pH 7.8) containing 1% polyvinylpyrrolidone, and centrifuged at 15,000× *g* for 20 min at 4 °C. The supernatant was taken to measure the activities of SOD and POD, and absorbance was recorded at 560 nm and 470 nm, respectively. The absorbance of the samples was measured by a SmartSpecTM Plus spectrophotometer (BioRad, Hercules, CA, USA). Fifteen plants were pooled together as one sample (≥1.5 g) in each replicate. Five biological replicates and three technical replicates were performed at each time point.

### 4.3. Multiple Phytohormone Profiling

The content of ABA, JA, ACC, IAA, SA and tZ in leaves of 93-11D and 93-11T were determined at 0, 3, 6, 12, and 24 hpt. Briefly, leaf samples (50 mg) were ground with liquid nitrogen, the chloroform was added to separate the different phases, the sublayer mixture was subsequently re-suspended with pre-chilled 80% methanol. The supernatant was assayed using an ACQUITY UHPLC system (Waters Corporation, Milford, MA, USA) coupled with an SCIEX Triple Quad™ 5500+ LC-MS/MS system (AB SCIEX, Framingham, MA, USA) [49]. All the processes were performed by Lu Ming Biotechnology Co., Ltd. (Shanghai, China). Three biological replicates were carried out, and each biological replicate was run in three technical replicates.

### 4.4. RNA Isolation, RNA Sequencing (RNA seq)

Leaf and root samples were collected at 6 hpt from the trefoil stage of 93-11D and 93-11T. Tissues without (control) or with Na_2_CO_3_ treatment (three biological replicates, 24 samples in total) were sent to the Biomarker Technologies Company (Beijing, China) for RNA seq. RNA seq was performed by combining TGS, which derived transcriptome data to assemble a full-length transcript for structural optimization, and NGS which was applied for transcriptome data correction as well as quantitative analysis of the transcripts from TGS. Total RNA was extracted by TRIzol method (Life Technologies Invitrogen, Carlsbad, CA, USA) according to the manufacturer’s protocol. RNA concentration, purification, absorbance of nucleic acids and integrity were assessed on a NanoDrop 2000 (Thermo Scientific, Waltham, MA, USA) and Agilent 2100 Bioanalyzer system. The prepared libraries were sequenced on the Illumina HiSeq Xten and PacBio RS II platform for NGS and TGS, respectively, to generate pair-end raw reads. The raw reads were firstly processed through in-house perl scripts and filtered to exclude adapters, reads containing poly-N and low-quality reads after data processing. At the same time, Q20, Q30, GC-content and sequence duplication level of the clean reads were calculated. All the downstream analyses were based on clean reads with high quality. These clean reads were then mapped to the reference *Oryza sativa* ssp. *japonica* cv. Nipponbare genome (https://rapdb.dna.affrc.go.jp, accessed on 5 November 2021) using Tophat2 tools soft. Gene expression levels were estimated by FPKM (fragments per kilobase of transcript per million fragments mapped). DEGs were identified by TBtools (version 1.09866) based on adjusted *p*-value < 0.05 and |log_2_ (fold change)| > 2. The adjusted *p*-value was obtained by the Benjamini and Bonferroni correction approach for controlling the false discovery rate. The GO functional classifications and KEGG pathway analyses were conducted using the 2021-updated DAVID [99].

### 4.5. Quantitative Real-Time Polymerase Chain Reaction (qRT-PCR)

Total RNA used for qRT-PCR was extracted as above described. First-strand cDNAs were synthesized from approximately 2 μg of total RNA with gene-specific primers (Supplementary Table S11). The primers were downloaded from qPCR Primer Database (https://biodb.swu.edu.cn/qprimerdb/). The qRT-PCR amplification was conducted in a volume of 20 μL containing 10 μL of 2× *TransStart*^®^ Top Green qPCR SuperMix using a Top Green qPCR SuperMix kit (TransGen Biotech, Beijing, China). Each sample had three technical replicates and normalized to the reference gene GADPH (Supplementary Table S11). 

### 4.6. Statistical Analysis

Pearson’s correlation coefficient was used to measure the linear relationship between two variables and calculated by R statistical package. The heatmap was generated according to the correlation values or gene expression levels, and plotted by https://www.bioinformatics.com.cn (accessed on 18 December 2021), a free online platform for data analysis and visualization. The data of inhibition rate were analyzed by *t* tests. The other physiological data was analyzed by one-way analysis of variance (ANOVA) followed by Duncan’s multiple range test. Data are represented as the mean ± SE (standard error) of three or five biological replicates. Data visualization was carried out in GraphPad prism version 9.0 (GraphPad software, San Diego, CA, USA), or Microsoft Office Excel, taking * *p* < 0.05 and ** *p* < 0.01 as significance.

## 5. Conclusions

In conclusion, the saline-alkali tolerance of tetraploid rice compared with diploid rice is mainly manifested in its higher activities of POD and SOD, relatively high SA levels and low IAA and their positive correlation, as well as the activations of the lignin biosynthesis pathway and wax biosynthesis pathway. This study provides excellent gene resources and valuable references for genetic breeding to improve saline-alkali tolerance of crops.

## Data Availability

The datasets generated and analyzed in this study are available at PRJNA812638 (https://www.ncbi.nlm.nih.gov/sra/PRJNA812638, accessed on 5 May 2022), as well as PRJNA873237 (https://www.ncbi.nlm.nih.gov/bioproject/?term=PRJNA873237, accessed on 26 August 2022).

## Supplementary Materials

The following supporting information can be downloaded at: https://www.mdpi.com/article/10.3390/ijms232416146/s1.
