# Supplementary material for: Comparative Analysis of Physiological, Hormonal and Transcriptomic Responses Reveal Mechanisms of Saline-Alkali Tolerance in Autotetraploid Rice (Oryza sativa L.)
Source: Int J Mol Sci. 2022 Dec 18;23(24):16146. doi: 10.3390/ijms232416146 (PMC9783840; doi:10.3390/ijms232416146)
Supplement: Supplementary file 1 [file ijms-23-16146-s001.zip › Supplementary Legends.pdf]

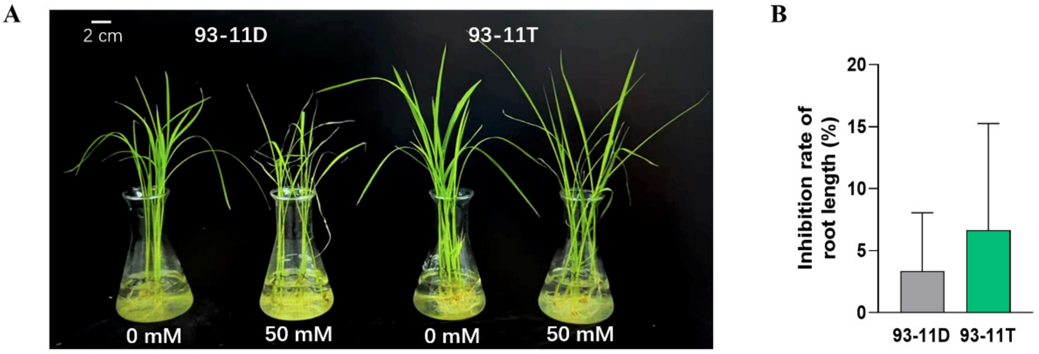

Figure S1. The Enhanced saline-alkali tolerance in 93-11T autotetraploid rice. (A) Phenotypic changes of 93-11D and 93-11T under control or 50 mM Na_2CO_3 treatment conditions for 24 h, scale bar = 2 cm. (B) Inhibition rate of root length was measured at 7 d after 50 mM Na_2CO_3 treatment. Error bar indicates standard error (SE, *n*=7).

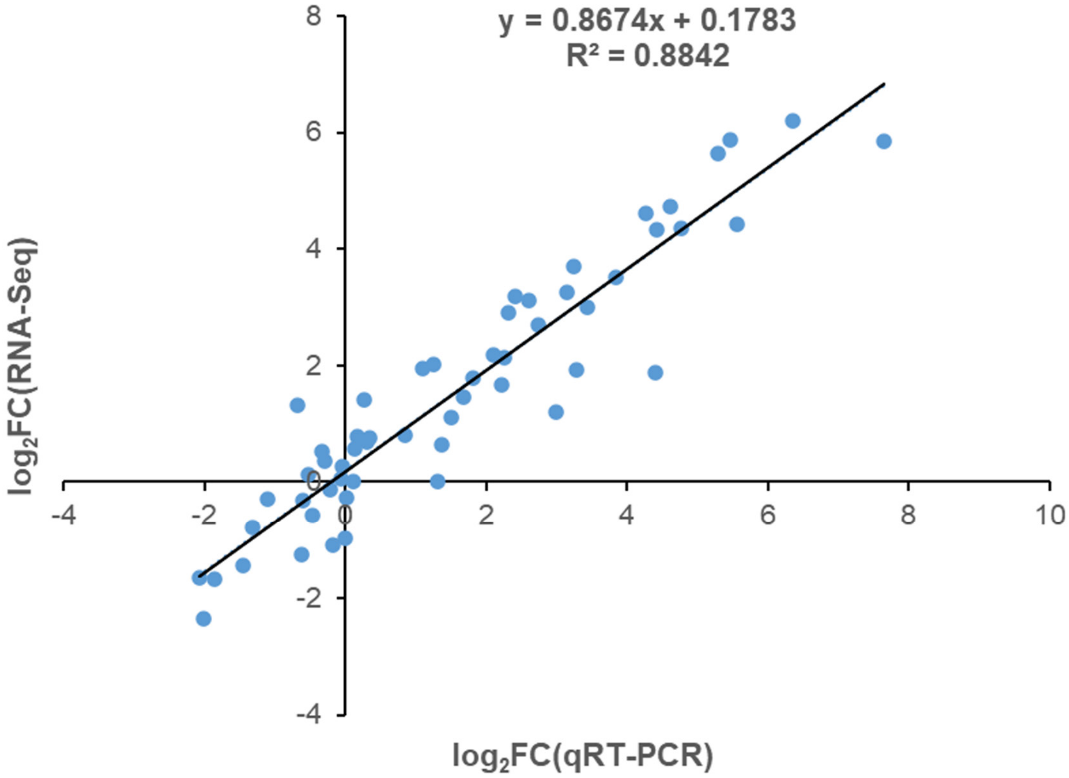

Figure S2. Correlation between the fold change (FC) analyzed by RNA-seq (*Y*-axis) and data obtained using qRT-PCR (*X*-axis). The expression of eight genes in DC, TC, TS and DS of leaves and roots for validation of the transcriptome results. The reference line represents the linear relationship between the results of RNA-seq and qRT-PCR. DC and TC represent diploid and tetraploid without stress, respectively; DS and TS represent diploid and tetraploid under Na_2CO_3 stress, respectively.

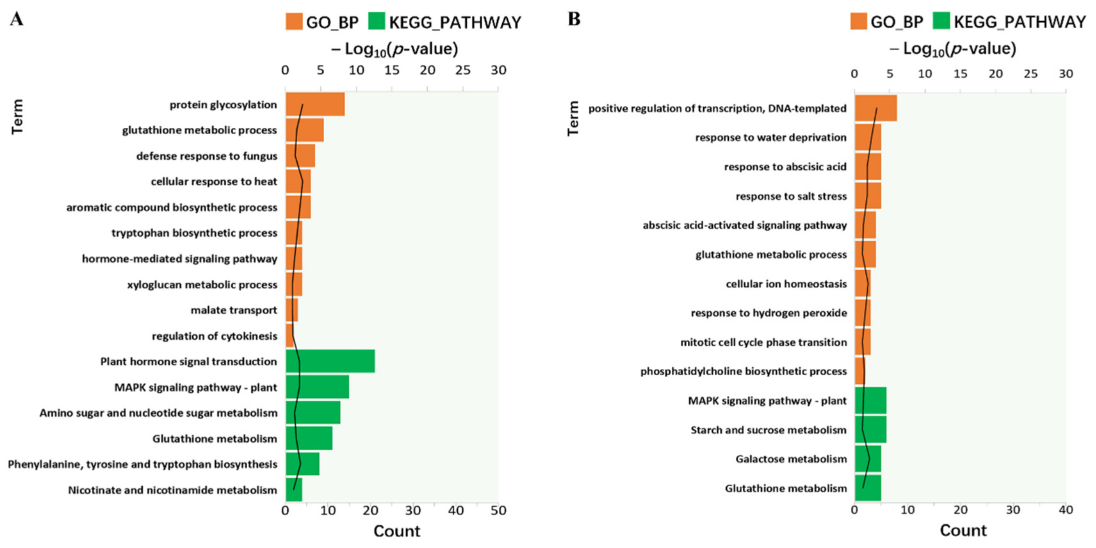

Figure S3. GO functional and KEGG enrichment analysis for trend DEGs in 93-11D and 93-11T under Na_2CO_3 stress (TS vs. DS). Downtrend genes in roots (A), and leaves (B). The black line represents $-\log_{10}$ (*p*-value), refer to the upper scale; the number of enriched genes refer to the bottom scale; orange column represents GO_BP, green column represents KEGG pathway. GO: Gene Ontology; KEGG: Kyoto Encyclopedia of Genes and Genomes; BP: biological processes; DEGs: differentially expressed genes. DS and TS represent diploid and tetraploid under Na_2CO_3 stress, respectively.
